# Seed credit model in Uganda”: Participation and empowerment dynamics among smallholder women and men farmers

**DOI:** 10.1016/j.gfs.2023.100720

**Published:** 2023-12

**Authors:** Grace Nanyonjo, Eileen Nchanji

**Affiliations:** a National Crops Resources Research Institute (NaCRRI), Kampala, Uganda; b International Center for Tropical Agriculture, Nairobi, Kenya

**Keywords:** Gender dynamics, Seed system, Empowerment, Decision making, Seed credit model, Food security

## Abstract

Seed is life and can be a source of empowerment and disempowerment for women and men farmers. In this study, to close the gender gaps in seed, the Community Enterprises Development Organization, the Alliance of Bioversity and CIAT and the National Agricultural Research Organization developed a seed credit model available to men and women belonging to farmer groups. A mixed method was used to collect information from two districts in central Uganda on how the seed credit model reconstructed access, use, control and resulting benefits. Results showed that the provision of the seed credit model was considered a blessing even though it had many nuances. As a result of the seed credit model, we saw increased productivity in women's fields, increased income and decision making over income incurred from the sale of their crops. Their social status has been enhanced, and they now occupy a place of respect in their communities and households, where they can make decisions and get assets like houses and land. While it increased productivity, income and enhanced food and nutrition security needs of the family, it also changed power dynamics within the household as women become more empowered. To maintain power relations, men limited women's access to fertile land and family labor, which defined the quantity of seed gotten from the seed credit model. Women's participation and involvement in the seed credit model decreased over time as they were expected to pay their spouses' seed loans. Men's participation decreased because they were no longer entrusted with seed loans as their payment rate was very low. As we reap positive benefits, we have to ensure we don't ‘do harm’ when empowering our beneficiaries.

## Highlights

• Women and men's participation in the seed credit model decreased over time.
• Women were able to increase productivity, income and assets from the SCM.
• Gender based violence became prominent with changing power relations, as women got more empowered.
• Men were no longer entrusted with seed loans due to the low rate of repayment compared to women.

## 1 Introduction

Common bean (*Phaseolus vulgaris*) plays a fundamental role in sustaining the livelihoods of many rural and urban households through improved food and nutrition security in Eastern, Central, and Southern Africa (Buruchara et al., 2011). High in protein and insoluble fibre (Larochelle et al., 2017), beans represent the most affordable and dependable staple food among the rural and urban poor. In Uganda, the role of women in bean production is crucial as they control most of the production (Kiwanuka et al., 2020; Vernooy, 2016). However, limited access to good quality seeds has hindered their ability to meet the increased demand for home consumption and market (Aseete et al., 2018). Studies have estimated that 60–80% of the seed used by smallholder farmers in developing countries is home saved and obtained through informal distribution channels (Vernooy, 2016). Women farmers play a vital role in the seed system, especially through community seed banks, even though they are often ignored by researchers, development personnel, policies, and programs (Vernooy, 2016; Sahoo et al., 2018). Khan et al. (2016) mentioned that women's decision to acquire and use seeds is critical because they are considered seed custodians and household managers. It has been noted that marginalized women farmers encounter an acute shortage of improved quality seeds in an attempt to maintain household food and nutrition security. Quality seed in this study refers to any bean seed with good germination or regeneration ability, desirable moisture content, true to the mother population, uniform, and free from disease-causing organisms and insect pests (Sahoo et al., 2018). Seed use and control in this study refer to women and men farmers' ability to decide on the seeds to the sourced, when, and how to use the associated benefits and income. The existing seed systems have been renowned for ineffectively relaying improved seed to farmers, especially last mile farmers. The informal seed system, most common to women, doesn't quickly dispatch improved varieties generated (Hanif and Sperling, 2017), while the formal neither reaches women nor the last mile farmers.

Mudege et al. (2016) reported that fundamental gender and cultural norms determine who gets access to information on where and what prices of quality seeds. The unavailability of improved bean seeds in local shops makes access further impossible for women whose mobility is restricted (Kramer and Galiè, 2020). Yet if women and men had equal access to agricultural inputs, including improved quality seeds, sustainable bean production would be achieved (Miriti et al., 2013) with positive outcomes at the individual and household levels (Johnson et al., 2016). Similarly, gender norms influence decisions on seed access and availability (Sahoo et al., 2018; Mudege et al., 2016).

Power relations and socio-cultural norms at various institutional levels further influence women's ability to access, use and benefit from quality seed. Mudega et al. found that women had a low level of participation in decision-making processes, limited access to finances, low awareness of potato seed systems, and were not considered farmers but just assistants to farmers, who are the men. Amri (2010) further notes that decision-making varies across crops and varieties in line with market orientation. For example, women in Tanzania and Ethiopia controlled food crops but not cash crops. Socio-economic and cultural challenges in agricultural production further perpetuate inequalities in the distribution of productive resources, including improved quality seeds (Nkenga et al., 2020). With the commercialization of beans and men's taking over the market, the existing social structures will further hinder women's access to agricultural inputs (Harman Parks et al., 2015). Hence, breaking the structural barriers and harmful gender norms impeding access to improved quality seeds will ensure women's full participation and benefit from any seed distribution system (Kramer and Galiè, 2020).

A number of development partners and researchers have advocated for inclusive, gender-responsive, and innovative legume-centered seed distribution models in collaboration with the private sector to reach all farmers with better quality seeds and varieties (Buruchara et al., 2011). These seed distribution models address unfavorable norms and unequal access to improved seeds (Bossuet, 2020). The seed distribution models developed to channel improved legume seeds to farmers have only supplied 1% of the total seed (McGuire and Sperling, 2016). Plant breeding programs compound seed distribution issues by focusing on breeding crops with high value productivity and commercialization potential with traits that sometimes disempower or exclude women (Mutari et al., 2021). Some recently released seed varieties sometimes increase women's labor burden while also increasing the need for complementary inputs (Kramer and Galiè., 2020; Nchanji et al., 2021), all of which may be barriers for resource poor women farmers. In addition, when breeders interact with small-scale farmers, they interact primarily with men due to the social norms where men, as the head of the households, are gatekeepers (Sahoo et al., 2018). This makes them overlook the traits preferred by women farmers that move beyond food and nutritional security.

Currently, the Ugandan government is promoting innovations (public subsidies and voucher programs), prioritizing women's access to improved quality seeds; while private seed companies are now focusing on establishing demonstration plots managed by women in inaccessible locations alongside small packs of improved seeds to meet the seed needs of women (Kramer and Galiè, 2020). Nevertheless, integrating both formal and informal seed distribution systems promise to be gender-responsive innovation making use of social networks for the diffusion of improved quality seeds (Kramer and Galiè, 2020). Social networks promote farmer-to-farmer seed exchange, thus a reliable and trustworthy mechanism of seed access (Poudel et al., 2015). It is, however, not certain if the innovation could overcome specific gender barriers in seed access. Literature also suggests the need to look at issues beyond seed access and focus on how seed distribution models improve the use and control of quality seeds and the benefits arising from their use (Kramer and Galiè, 2020). However, there is scanty literature on how seed distribution models improve women's and men's decisions regarding seed use and control with associated benefits within the household (Kramer and Galiè, 2020). Studies conducted in Tanzania and Ethiopia show increased women's control over seeds for food crops rather than cash crops (Amri and Kimaro, 2010). Since beans are now commercialized, there is a need to prove this finding in a social context where beans are produced and utilized (Businge et al., 2020).

### 1.1 Context of the study

To ensure equal access to seed by all men and women farmers, a seed credit model (SCM) was introduced by the National Agricultural Research Organization (NARO) in collaboration with the Community Enterprise Development Organization (CEDO) among bean farmers in Uganda in 2015. In this arrangement, NARO provides CEDO with foundation seed which CEDO multiplies and distributes under SCM. Through the seed credit model, improved quality bean seed of different bean varieties is made available to women and men farmers in organized groups on credit, and they pay after harvesting. Because beans have a woman's face, the model aimed at empowering women farmers with easy access to affordable and appropriately improved bean seeds to take on commercial production. The model was complemented with agronomic training, pre-financing, access to fertilizers, and monitoring visits to women's and men's farmer fields. The model has been implemented in three districts of central Uganda to give women and men farmers an alternative seed source with resulting income. This model has reached more than 266 women and 190 men. However, since its launch in 2015, little is known how it has addressed access, availability, quality, and use of improved seed by women and men farmers. Also, differences in the participation of women and men farmers in the model are unknown. Notably, the extent to which the model reached, benefited and empowered women is also not known. Little evidence on the gendered aspects of seed systems calls for research to bridge knowledge and evidence gaps to inform seed systems interventions (Puskur et al., 2021). Hence, this informed this study to generate information on gender dynamics in SCM - through understanding experiences in the context of women and men farmers. Understanding the gender differences in seed access, availability, use, control, and sharing of the benefits is crucial for development programs focusing on reducing gender inequalities in the SCM. Thus, this study focused on the contribution of the SCM in reducing gender inequalities in seed availability, access, seed use, benefits and empowerment of women as grain producers, traders, and users. It is organized around three research questions: (i) How has the SCM facilitated reach, access and benefited women as seed producers, traders and users (ii) How has the SCM facilitated use and control of benefits from bean sale and women empowerment (iii) what the gender-based benefits and constraints to participation in SCM are.

## 2 Methodology and study area

### 2.1 Study area

**Fig. 1:**
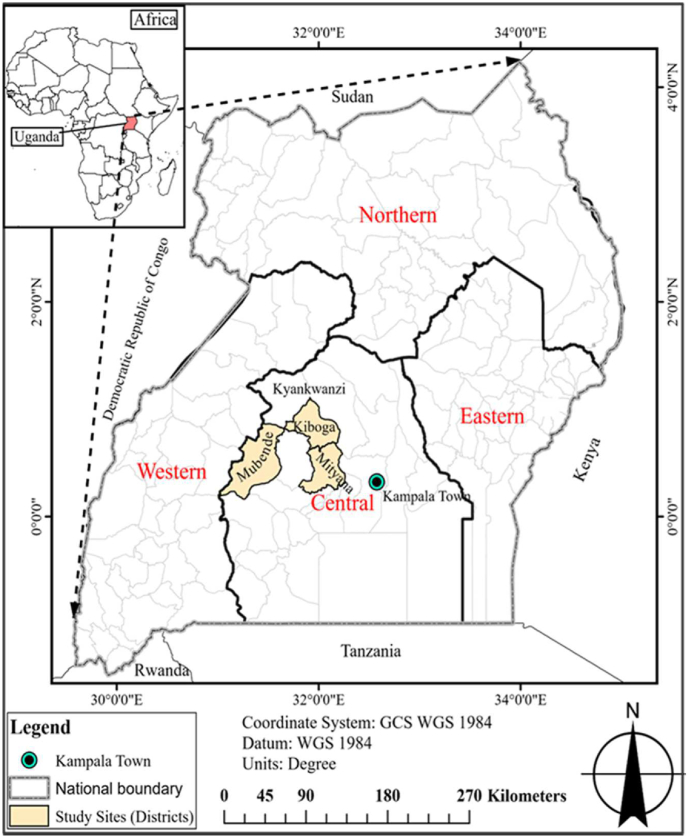
Map of study sites in Uganda.

The study was conducted in three districts of central Uganda, including Mubende, Mityana, and Kiboga as shown in Fig. 1. Agriculture is the main source of income for majority of the residents of these three districts. In particular, crop production is the primary form of agriculture in the region with most people participating in the cultivation of crops such as beans, maize, coffee, banana, cassava, groundnuts and sweet potato. Common bean is a priority crop in these three districts and is virtually grown by every household either for food, sale or both. Besides being an agricultural region, these districts were selected for the study because the seed credit model has been operational in the area since its inception in 2015.

### 2.2 Sampling procedure and sample size determination

Multi-stage random sampling technique was used to obtain an unbiased sample, ensuring the reliability and generalizability of the collected data. The three districts of central Uganda, namely Mubende, Mityana, and Kiboga, were purposively selected for the study. Within these districts, sub-counties were selected based on the availability of farmer groups using the seed credit model (SCM). Two sub-counties were chosen from Mubende, and one sub-county each from Mityana and Kiboga.

At the sub-county level, farmer groups participating in the SCM were purposively selected. The selection of these groups aimed to include respondents who had knowledge about the subject matter under investigation. Two groups were selected from Mubende, three from Mityana, and two from Kiboga, depending on the availability of groups participating in SCM. At the group level, member lists were obtained from the group leaders. From these lists, a total of 200 respondents (134 women and 66 men farmers) were randomly selected and interviewed. To account for small group membership, every 3rd person was randomly selected from the list. Initially, the study aimed to interview at least 270 respondents, with 90 respondents per district and 45 per sub-county. However, at the time of the study the sample size was reduced to 200 participants because some women and men farmers failed to consent to be interviewed.

The breakdown of the sample size by district and sub-county is as follows.

The study also conducted two sex disaggregated focus group discussions in each sub-county with participants from the selected groups. At each FGD site, preliminary meetings were held with the group leaders to introduce and explain the purpose of the study and its impact on the modalities of the seed credit model. The group leaders who had local knowledge of the area and participants of the SCM model helped the research team to identify men and women farmers who participated in the FGD study. The key selection criteria for participants of the FGD included (1) being a current participant of the SCM, (2) having experience with the subject under investigation, and (3) willingness and availability to participate in the discussion. FGDs held had 8-10 participants.

### 2.3 Data collection

#### 2.3.1 Surveys

Using a pretested questionnaire, quantitative data was collected from 200 farmers drawn from the three districts, of which the majority (67%) were women farmers and 33% men farmers. The questionnaire was administered to the selected women and men farmers by well-trained enumerators fluent in Luganda, the study communities' main language. The questionnaire was organized into sections. The first section collected data on the socio-economic characteristics of women and men farmers, access, use, decision making, and factors hindering or enhancing participation in SCM. The second section of the questionnaire collected information on seed availability. on the third section collected data on the dynamics in farm size to capture the differences in land accessed, bean field ownership, and management during active years (2015, 2016 & 2017) of participation and the seasons of 2020A & 2020B.

#### 2.3.2 Focus group discussions

This method was used to triangulate findings from the quantitative survey due to its ability to provide detailed accounts of human experience and a deeper analysis of social and cultural experiences, which this study examined. Hence, qualitative data was collected to explore the experiences of women and men farmers as participants of the Seed Credit Model. A trained research assistant conversant with Luganda language (central language) and a researcher conducted the FGDs. The researcher was the discussant and the research assistant a notetaker. Also, an audio recorder was used, and saved recordings were later transcribed and translated. With separate FGDs held with men and women, data was collected on knowledge/community perception about the SCM, seed access, use and control over other production resources, decision making, and determinants of participation intensity of women and men in the model.

#### 2.3.3 Key Informant Interviews

Preliminary meetings were held with the coordinator and chairpersons of the groups to introduce and explain the purpose of the study. They were selected as key informants because they had local knowledge of the area and participants in the SCM model, and gender dynamics in the community. The coordinator and chairpersons of the groups were interviewed. Questions focused on how the seed credit model was introduced to the community, the process of benefiting, how it was sustained and the gendered dynamics that resulted from this new seed credit model approach. Their answers were used to validate and triangulate data from FDGs and surveys and answer the why to give depth to the study.

### 2.4 Data analysis

**After data collection, analysis of the two data sets was done concurrently.** Quantitative data was cleaned and analyzed using the STATA statistical software package. The researcher preferred this package because it provided a more thorough and rigorous coding, interpretation, and enhanced data management. Descriptive analysis with chi-square tests was used to determine the gender differences in on-farm enterprise size, seed access, use and associated benefits of participation in SCM, and decision-making across the districts. Descriptive statistics such as means, percentages, standard deviations, and frequencies were generated. Almost all the analysis was disaggregated by gender and districts to show the difference and/or similarities. The recorded qualitative data were transcribed and translated into English for analysis. A thematic analysis approach was used to classify and analyze responses concerning gender differences in SCM. To explain the trends and the why drawn from the quantitative data (see Table 1).

## 3 Results

### 3.1 Socio-demographic characteristics

Table 2 presents the demographic characteristics of the participants. The results demonstrate a significant gender imbalance among the interviewees, with women comprising 67% of the participants compared to men at 33%. Most of the participants were aged between 30 and 45 years. However, in the Kiboga district, many of the women were within the age range of 46–64 years, while the same age range was predominant among men in the Mityana district. This discrepancy in age distribution is possibly explained by the specific targeting of groups where women were the majority, such as Key Informant Interviews (KII) and Community Enterprise Development Organizations (CEDO). According to similar findings by Sakho-Jimbira and Hathie (2020), the higher representation of women compared to men in the age range of 30–45 years across the three districts suggests that women play a significant role in agricultural activities, including labor and decision-making, in many rural areas. Furthermore, women were more accessible for interviews due to their predominant employment in agriculture, in contrast to men. Moreover, the women showed a greater willingness to participate in the study, resulting in a higher representation of women in the sample.

**Table 1:** Sampled sub-counties by district and corresponding sample size.

District	Subcounty	Female respondents	Male respondents	Total respondents
Mityana	Ssekanyonyi	58	20	78
Kiboga	Kibiga	19	09	28
Mubende	Kasanda	57	37	94
Total		134	66	200

**Table 2:** Showing socio-economic characteristics of women and men farmers across districts.

Demographic variables	Mubende			Kiboga			Mityana		
	Female	Male	$\chi_{1}^{2}$	Female	Male	$\chi_{1}^{2}$	Female	Male	$\chi_{1}^{2}$
**Age groups**									
15–29 years		17.1	12.41***	10.0		2.83	1.7	5.0	6.89
30–45 years	52.5	48.8		35.0	60.0		58.6	25.0	
46–64 years	42.6	26.8		50.0	30.0		34.5	60.0	
65 years & above	4.9	7.3		5.0	10.0		5.2	10.0	
**Marital status**									
Married (one spouse)	47.5	73.2	12.06***	50.0	60.0	5.48	41.4	60.0	11.41***
Single	3.3	2.4						10.0	
Separated	14.8			5.0	10.0		8.6	5.0	
Divorced							1.7		
Widow/Widower	13.1	2.4		35.0			18.9		
Married (polygamous)	21.3	21.9		10.0	30.0		29.3	25.0	
**Household type**									
Dual headed household	67.2	97.6	17.77***	55.0	90.0	7.73***	75.9	90.0	11.00***
Male headed households		2.4			10.0			10.0	
Female-headed household	29.5			40.0			24.1		
De facto female-headed	3.3			50.0					
**Educational level**									
None	1.6		1.6	10		3.5	5.2	15.0	2.6
Primary	65.6	60.9		55.0	70.0		60.3	55.0	
Secondary	29.5	31.7		35.0	20.0		31.0	30.0	
Tertiary	3.3	7.3			10.0		3.5		
**Household head**									
Yes	45.9	97.6	29.45***	50.0	100.0	7.5***	37.9	100.0	23.05***
No	54.1	2.4		50.0			62.1		

Majority of both men and women respondents in all the three districts were from dual households and were married to a single spouse even though a reasonable number of participants were in polygamous families. The prevalence of dual households and monogamous marriages in the three regions can be attributed to a combination of cultural, social, economic, and legal factors that shape marital and family structures in the region (Tumwine and Ntozi, 2017). Nevertheless, this demographic characteristic provides an additional lens through which to understand seed access, usage, and decision-making within participating households.

In the Kiboga district, a significant proportion of female respondents were either from female-headed households or de facto households. Within the de facto households, the women had visiting husbands who appeared only once or twice a year, potentially suggesting minimal male influence on household decisions. This highlights the unique dynamics within these households and underscores their potential implications for seed-related activities.

With regards to educational background of the interviewed farmers, both men and women had at least a primary education, although men had significantly higher years of education overall. This disparity in educational attainment may have an impact on men's greater participation in the model. In line with the findings of Nouman et al. (2013), who suggested that higher levels of education are associated with increased access to agricultural credit, it is plausible that the higher education levels among men in this study could be influencing their engagement in the model.

These findings shed light on how the interaction between gender, age, household structures, and educational levels of the interviewed farmers possibly influence seed access, utilization, and decision-making within households, and thus contribute to the broader understanding of agricultural practices in the studied districts.

### 3.2 Gendered participation in the seed credit model for the period 2015 to 2020

Fig. 1 presents the gender-disaggregated results for the years respondents have been active members of the seed credit model. Most of the participants in the first and second years (2015 and 2016, respectively) of the seed credit model were women. However, in 2017, the number of male participants surpassed that of women, with men accounting for 62% and women 48%. Nevertheless, the number of male participants started to decline in 2018 and continued to drop substantially in the subsequent years.

At the inception (2015), more women participated in the SCM than men because they received information about improved seed and SCM that enabled them to make an informed decision to join the model. This information was through the group chairpersons, and those who were not members by the time of inception obtained the information from friends who then belonged to groups. The majority of these are men who increasingly joined between 2016 & 2017 after witnessing better yields from friends' fields. A Focus Group Discussion with members of the Kamu farmers group confirmed that at the initial stages of the inception of the SCM, men were reluctant to join the initiative thus contributing to the low number of men compared to men in the first 2 years as demonstrated in Fig. 1.

*Before I joined this farmers' group, I used to see my friends and neighbors moving up and down the village, and when I asked them, they would say they were going for seed credit model meetings. My friends and I were not in these groups and had nicknamed those in the groups as village idlers, but when I later saw their yields, I quickly joined, and my life has never been the same* Male respondent, FGD, Kamu farmers group.

Other men who participated in the model were influenced and encouraged by their wives but only after witnessing the significant harvests at home and the availability of a ready market for the grain. Women participants noted that in the initial stages (especially the fast two years – 2015 and 2016), it was difficult to convince their husbands to participate in the seed credit model without seeing the benefits of those participating. However, with remarkable results from the project, men were convinced of the benefits of the project and thus joined en masse in the late 2016 to 2017. This explains the high number of male participants in SCM in 2017.

The benefits motivated men to participate in the project as demonstrated by the increased number of men in the project in 2017. Nonetheless, women still had more plots under beans compared to men despite men having access to more land than women (Table 3). However, a significant decline in men's participation was observed after 2017, as indicated by the data presented in Fig. 1. The reasons behind this decline were explored in Focus Group Discussions (FGDs), where participants highlighted the failure of the Seed Credit Model (SCM) to meet their immediate needs. These needs included access to quality seeds, a reliable local market, and prompt payments. Participants mentioned that these challenges posed financial burdens, making it difficult for them to sustain their involvement in the SCM. Much as improved quality seed improves yield potential and profitability of any farming effort, access to such seed is largely shaped by gender roles (Puskur et al., 2021; ISSD, visited on March 28, 2021). Therefore, women and men farmers experience diverse levels of satisfaction with the seed accessed (Puskur et al., 2021), and once not satisfied, some will opt out.

**Table 3:** Land accessed and plots under beans disaggregated by gender.

	Sex		p-value
	Women	Men	
Plots of Land Under Beans	1.49	1.35	0.020
Total Land Accessed	5	5.77	0.000

### 3.3 Seed credit model approach and framework

Data from FGD shows that all respondents understood the seed credit model as an arrangement introduced by NARO where identified pre-existing groups were registered to be beneficiaries of improved quality seed. Women and men said NARO and CEDO called up meetings where the arrangement was communicated, and they were taken through how it would work. Across all study sites, women and men understood the seed credit model as a contractual arrangement where the seed is loaned to a group, not individual farmers. Members of such groups are recognized by registration and payment of membership fees, annual subscription, and being part of the group savings scheme. Under the arrangement, group leaders signed contracts with CEDO upon receipt of the seeds. Women and men from FGDs pointed out that specified in the written contract was the seed price of UGX.4000 per kg, mode of payment, sorting of bean grain before the sale, and CEDO buying the grain of only supplied varieties either at UGX. 2000 as the bare minimum or any price not lower than the prevailing local market price per kg of grain. Also, a verbal contract was made with members on non-intercropping of the CEDO seed with local or other varieties and no late planting. To maintain non-intercropping, the chairpersons of various groups inspected their group members' fields before and after sowing to monitor progress and offer advice. Both men and women agreed with this understanding of SCM.

Under the arrangement, seeds were distributed to men and women farmers on credit depending on the request made and payback after harvesting. Men and women farmers further said they did not return physical money but rather when they were selling their harvests; CEDO usually subtracted the amount equivalent to the seed accessed. For example, if one was loaned 50 kgs of seed, one is expected to return 100 kgs of the equivalent. In case of poor harvests due to weather changes, farmers were usually excused from paying back seed credit irrespective of gender.

### 3.4 Community perception of the seed credit model

A larger section of women and men farmers perceived the seed model as a “blessing” that came to address the seed unavailability many faced in the past. The seed credit “blessing” answered both seed availability and quality challenges. Even without money, one could be assured of seed in required quantities which wasn't the case before. However, some respondents perceived the SCM as a mixed blessing, as they now had to deal with delayed payment and drudgery from spending more time sorting and grading beans to the aggregator's taste. This was mainly identified with men farmers who participated in the study.

The good quality improved seed required more attention than the local bean seed. For instance, one had to plant in rows and not broadcast, spacing on sowing was followed, weeding, applying fertilizer, spraying pesticides, and sorting the harvested beans to remove unwanted coloured beans, spoilt beans, and other dirt before they were sold. The sorting activity is perceived as tedious and labor-consuming, which has increased the workload on women interested in commercialization by the arrangement. These women provide sorting labor for their produced seed and that of their husbands (FGD). This comes when women already work more than 8.9 h than men in agriculture (Aryal and Kattel, 2019), yet the repetitive reproductive roles that consume much of women's time have not changed. As a result, women are constrained to work 16 h daily to balance competing needs in agricultural production, reproductive roles, and income generation (Bhimani and Desai, 2015). Since women provide over 60% of the agricultural workforce, the extended working hours expose them to musculoskeletal injuries and diseases (Singh and Singh, 2019). Hence a likely impact on bean productivity if nothing is done to reduce drudgery when women and men increase acreage due to improved availability and accessibility of improved seed.

### 3.5 Access to quality improved seeds

**Fig. 2:**
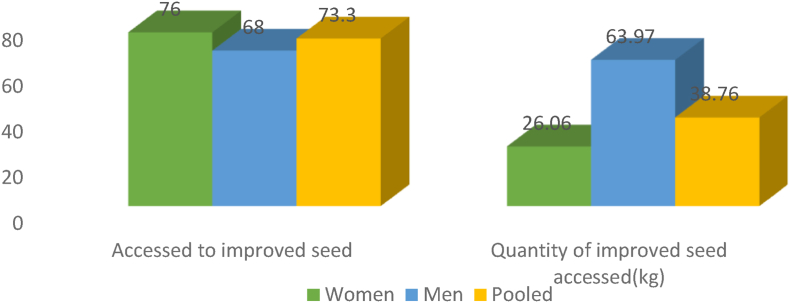
Access to and quantities of improved seed accessed by women and men farmers.

Fig. 2 shows the number of women and men farmers that accessed improved seeds during years of active participation in SCM. Though more women (76%) accessed improved seed than men (68%), quantities of seed accessed by men were substantially higher (Fig. 2). In this study, access was referred to as the ability to acquire seed in required quantities.

**Fig. 3:**
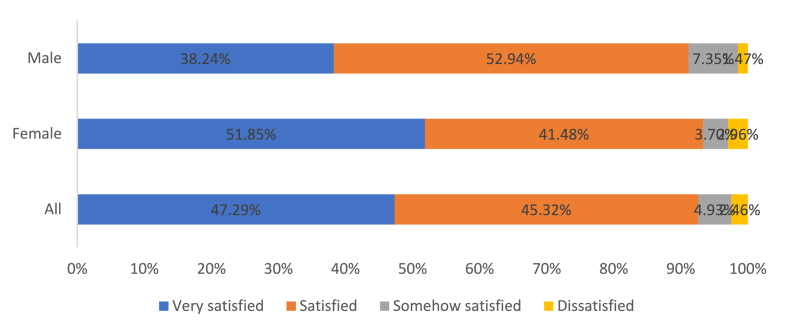
Satisfaction levels on seed accessed under SCM by women and men farmers.

From FGD, men and women farmers reported having an equal opportunity to access the seed even though the quantities of seed accessed were determined by factors such as the ability to pay back, and size of the garden owned. While more women than men had access to improved seed, men on average received more quantities of improved seed than women. This can be explained by the higher number of acres of land owned by men than women as demonstrated in Table 3. Men accessed more quantities of bean seed (34.8 kg) than 26.06 kg women accessed due to the land size. Group chairpersons monitored acreage prepared for bean production before issuing the requested seed amount. This was to confirm the size of the plot one owned. However, group leaders would make adjustments if CEDO supplied less stock to the group and priority was given to those who reliably paid their seed loans. Land ownership was one of the major determinants of the quantities of improved seed accessed by both men and women. However, on average there were more women having access to improved seed than men. According to the group leaders, women farmers were more honest and reliable in credit payback than men. This partly explains why more women either accessed the same quantities of seed required (81% women vs 56% men) or more than the required quantities (women 2% vs men 0%) (Fig. 3). This created problems in the household as women who benefited continually were alleged by their husbands to be engaging in extramarital affair with group leaders to imply that this is the reason why the group leaders offered them seeds in return as a token of appreciation. A focus group discussion by Kamu farmers group affirmed this notion.

“*Men fear paying back loaned seed claiming a lot of family responsibility which costs other group members. So in instances where the seed is not enough, we as leaders prioritize women because they are honest in paying back" male group leader, Kamu Kamu farmers group, Bukuya*

The seed was given to individual group members during seed distribution, and each signed for the quantities received. This also applied to couples in the same group as long as they reported different bean fields. A husband and wife each signed for individual quantities accessed. However, for couples working together, either wife or husband signed for seed received, and, in most cases, it was the wife to sign. This is because some men didn't want to pay back loaned seed, so they used their wives to sign for the seed acquired, and in case of failure to pay, it was none of the man's business, which means that the wives would suffer paying back the loans of their husbands. This frustration has caused many women to decide to have separate bean plots from their husbands to access credit and pay back with visible benefits.

Farmers, both women and men, from all study sites, perceived the Seed Credit Model (SCM) as a means of ensuring seed security. Under this model, they had guaranteed access to high-quality improved seeds, which would have been otherwise difficult to obtain without the credit arrangement. Access to these improved seeds enabled women and men farmers to increase bean productivity, leading to better yields and sufficient grain for food. Consequently, a higher percentage of women (69%) availed seed credit every season compared to men. Their aim was to achieve better yields by planting high-vigor seeds. However, a few men also sought seed credit regularly to switch to bean varieties with a local market. Such bean varieties facilitated independent selling by men without relying on external buyers like CEDO.

According to survey results (Fig. 3), most women (51.85%) expressed high satisfaction with the seed quality, while most men (52.94%) reported being satisfied. Dissatisfaction with seed quality was minimal (4.7% for men and 9.6% for women). Women and men confirmed that they had access to seeds even without money during the planting period. If they hadn't been part of the seed model, they would have faced the same issue as fellow farmers who continually reuse the same local seeds, compromising their harvests.

The varieties distributed under the model are said to be the right varieties capable of addressing gender needs, priorities, and challenges because of their gender friendly traits. Women and men, farmers, characterized the varieties accessed as high yielding, weather-resistant, and compatible with their soils than the indigenous local varieties. However, one variety known as NAROBEAN 1 was singled out as gender-responsive and a “miracle variety “embraced across all study sites as a non-disappointing variety that gave farmers high yields.

*“Imagine! I planted one kilogram of NAROBEAN 1, and I harvested 20kgs. When my friend told me it was a very profitable crop, I decided to test this variety. I can tell you that we have never benefited or profited in any crop the way we profited from NARO BEAN 1 since we were born"* female respondent, FGD, Kirangira farmers group

Participants reported that the seed credit scheme is a “blessing” to them that enhanced their access to improved seed with high yielding traits changing their lives as testified;

*“ … CEDO through NARO has helped us so much and has lifted us from very far; we had nowhere to run to and borrow like 50 kg of seed. It is only we who belong to a group that have the seed. Those who are not in a group do not have access to such good seeds. When they get from group members secretly, that is their arrangement and not group concern in case they are not paid back. As for me, everyone in this village knows me as the NARO woman because I always have good seeds and good harvests"* female respondent, FGD, Kamu kamu farmers' group, Bukuya, Mityana District.

This is how they described how women and men farmers understood seed distribution under SCM via local seed (Table 3). Women and men, farmers, perceived improved seed as quality seed that addressed the persistent challenges faced over time. Such challenges included climatic changes that increased the prevalence of pests and diseases, leading to low yields and food insecurity. But with bean seeds that are weather and disease resistant, high yielding, quick to cook, nutritious, and tasty, women and men farmers affirmed in FGD that their production, economic and social challenges were solved courtesy of their participation in SCM. Both men and women added that “easy to cook” beans have reduced quarrels and violence at home over foods that take longer to cook. They also attested that the economic, social, and health benefits of the composed varieties accessed were the major factors for the uptake of the credit model.

### 3.6 Availability of quality improved seeds

**Fig. 4:**
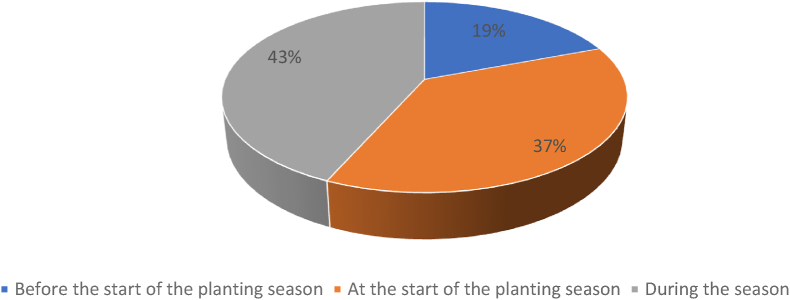
Period of the season when women and men farmers access seed through the seed credit model.

Availability of quality improved seed is the ability of women and men farmers to get seed from the right place and at the right planting time (Kramer and Galiè, 2020). A consolidated quantity of seed was delivered to respective group stores or chairperson premises by CEDO; these chairpersons then distributed it to women and men farmers. The mean distance to the chairperson's premises was said to be 1–2 km, accessible enough to accommodate women farmers with restricted mobility or lack of transport means to access the seed. The survey results indicate that the majority of the farmers, irrespective of their sex, have been able to access bean seeds either before the start of the planting season (43%) or at the start of the season (37%) (Fig. 4). According to Puskur et al. (2021), timely access to seeds is critical for farmers as it influences harvests and related decisions.

Women farmers from FGD indicated that having bean seeds before the season enabled them to plan for labor as they had to work on their husbands' plots. It also helped them to know how much land to hire for bean production. Thirdly, having bean seeds on time enabled them to plan early for better yields.

### 3.7 How to reach, benefit and empower women and men through seed

#### 3.7.1 Seed use and decision making

Of the seed quantities accessed, women farmers did not plant all the seeds. This is because most women shared the SCM seeds with other women who could not join the groups due to either restriction by the husband or failure to meet the required group criteria. This practice is common with home saved seed, which women share with relatives, friends, and neighbors who cannot access seed in a given planting season. This means that the SCM has not prevented women from providing seed bails to fellow women. Secondly, other women reported not planting all the seeds due to issues of limited land amidst competing crops. Whereas men planted all the seeds, they accessed. This could be because very few men would lack land to produce any crop of their choice (Doss et al., 2015).

At the household level, decision-making was a challenge among the married respondents. Women who were widowed reported making decisions together with their children concerning acreage to plant, the amount of seed to apply for, and how much of the final product to sell and keep at home. Similarly, women whose husbands were engaged in different crops like coffee or maize took independent decisions on bean production, varieties accessed, the quantity of seed to apply for, how many acres of land to hire, and when and how to weed, spray and harvest. The men only sold the beans produced by their wives, alleging that women are busy with household chores and, therefore, cannot engage with the buyers (FGD). Conversely, survey results showed that more women (81%) decided when to sell their bean crop than men (72%).

However, the decision to spend money was partially independent as 80% of women reported controlling their income compared to 79% of men. This indicates that access to improved quality seeds improved women's confidence to make decisions to better their lives, as evidenced in India and Bangladesh. Also, joint decision-making by husband and wife was more evident (21%) in the income obtained by women compared to that of men (18%). Secondly, no woman was observed making final decisions on the income earned by husbands compared to 2% who made final decisions on the use of income earned by their wives. Women's independence in making final decisions on the use of income shows the transformation in gender relations, even though joint planning failed in some homes, as reported by some women (FGD). They claimed their husbands were never open with how they spent their income. In one of the districts, women confessed to having witnessed many cases where they or their close relatives had gone through unbearable stress, having been disappointed by their spouses and in-laws. The disappointment came after men sold women's produce and used all the money without women's consent.

These experiences have opened their eyes to prepare for any eventualities. In the female FGD, one respondent said;

*“Men here have different interests from women. For them, they work hard to marry many wives. Yet, for us, we are looking at home development, like buying saucepans, mattresses, bedsheets, and children's clothes. We have issues with joint planning with our husbands. You work with them, and after many years he chases you, you start from scratch. If he dies, relatives start asking you if you have no place where you were born. This is why we want to have private property in preparation for these scenarios"* Female FGD, Agaliawamu farmers group, Kittanswa

#### 3.7.2 Benefits of participating in the model through enhanced knowledge and production skills

Both women and male respondents reported access to free agronomic training on managing improved seeds. Also, drawing experiences from each other, including learning visits to colleagues with better bean fields - women and men farmers in FGDs confirmed enhancing the productivity of their bean fields. The need for “family planning” (call it proper spacing) in beans, as echoed by women in FGDs, led to better yields, even though it increased the workload for women. This was a new technology learnt and adopted under the SCM. Such incentives that enhance women's knowledge and skills are believed to increase women's use of improved seed (Puskur et al., 2021), a key production input for maximum productivity.

#### 3.7.3 School fees relief

Both men and women respondents hailed their participation in the seed credit model for enabling their children to go to school. They said being part of the credit model acted as school fees security. Once their bean grain was bulked at the aggregation centres, it was easy to convince the school headteachers when payment was due. The school headteachers would visit the aggregation centres to get assurance from the group chairpersons on the grain availability and the possibility of getting school fees paid by the group members. On the other hand, some farmers exchanged the equivalent of bean grain with the school's demands. With these arrangements, the seed credit model participants never had their children sent away from school for school dues. As put by one middle-aged woman;

*“For me, even when CEDO delays the pay, my children were never chased from school. When the headmaster asks for fees, I refer him to discuss with the chairman of the group, who assures him that I am about to get good money and will be able to pay for my children. Sometimes I even show the headteacher my harvests, and he gets an assurance that I will pay …. even women who would not have beans would use our bean store as school fees security until they get money to pay fees" Female, FGD, Kamu Kamu farmers group*

The school fees excitement did not come with women only. Many men also mentioned that they had been relieved of the school fees burden and fulfilled their dreams.

*“At first, they gave us many seed varieties, and we never got good yields until we identified that NAROBEAN 1 works miracles for us … for me, my dream was to educate my children, and I am happy that they have all studied from beans only, now they are home due to covid 19″* male, FGD, Kamu Kamu farmers group.

#### 3.7.4 Economic empowerment and sharing of roles

Though some men expressed disappointment in their wives for not spending their earned income on household needs, other men were excited that their wives had relieved them of household responsibilities. Women farmers also mentioned that they gained fame and respect in their respective communities ever since joining the SCM. Many are perceived as responsible citizens who have taken on a profitable venture that requires hard work but, at the same time, good money.

**Fig. 5:**
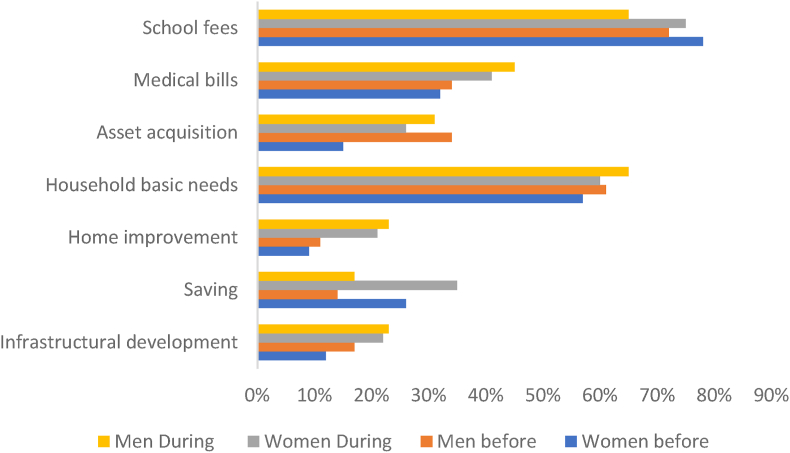
Women and men's expenditure details on the use of their income from bean sales before and after joining the SCM.

**Fig. 6:**
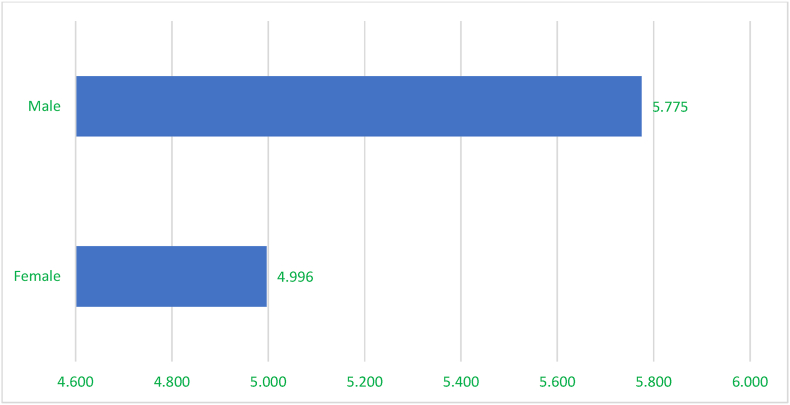
Average acres of land accessed disaggregated by gender (*p* < 0.000).

Surprisingly, the delay in payment was seen as a ‘blessing’ to many men, as it helped them earn lump sum money which they said helped them to invest in development ventures like building houses, payment of school fees, buying plots of land, and marrying more wives (Fig. 5). The delay in payment for women made them save money for emergencies as they waited for the pay. They added that lump sum payments enabled them to acquire some assets (Fig. 5) without their husbands knowing as a fallback in case of marriage failures (see Fig. 6).

#### 3.7.5 Autonomy in bean production

Most couples acknowledged that they had previously attempted to have joint gardens, but this endeavor often led to significant conflicts. Couples would struggle to reach agreements on various decisions, particularly regarding the sale of the harvest and the allocation of the proceeds. Women reported that men typically controlled and distributed the income generated from selling bean grain from the joint garden. Men would decide how the money should be spent, including expenses such as school fees, land hire, and a few household necessities. Unfortunately, women's needs were often overlooked, as the remaining balance was typically considered as general family expenses.

However, with their participation in the Seed Credit Model (SCM), which ensures access to quality seeds, women made the decision to have separate bean plots on rented land. This allowed them to earn their own income and fulfill their unmet needs. Although men generally preferred joint production, women added that having separate bean gardens saved them from conflicts regarding the division of the remaining balance.

Moreover, women expressed a preference for locating their gardens far from their homes to avoid constant supervision and monitoring by their husbands. By doing so, they could independently manage their farms and plan accordingly for the income generated from their produce. Women emphasized that if their husbands were unaware of the quantities of beans harvested, they could sell the produce and use the money to invest in their personal assets. Therefore, joining the seed credit model was a significant turning point, enabling them to benefit from their hard work, which was not the case previously (as mentioned in the focus group discussions, or FGDs).

## 4 Gender-based constraints to participation in the model

### 4.1 Failure by men farmers to repay loaned beans

Since CEDO seeds loan to groups, not individual members, information from KIIs and FGDs said men had challenges repaying the loaned seed. On the contrary, survey results showed that more women (16%) than men (3%) faced challenges repaying the loaned seed. However, poor harvest was the most cited challenge leading to repayment failure; women farmers, unlike men, attributed it to the competitive household needs. This observation comes when gender roles are shifting, with women increasingly substituting men's social roles (Nanyonjo et al., 2020). Even with all these challenges, women farmers always found ways to repay the loaned seed by borrowing money from savings groups. While for men, the burden of repaying the loan fell on other group members who at times were spouses to such men. According to the chairpersons of some farmer groups, many men sold their bean grain at the side markets and failed to repay loaned seed. These men were considered untrustworthy and impatient, but they defended the act by claiming they had a lot of family obligations at home. Such obligations necessitated them to have money at all times, forcing them to sell their produce secretly to other buyers. When bean grain is not sold through the group channels, it becomes hard for group leaders to claim seed repayment from farmers (KII). Most men said they used the money to pay school fees for their children, medical bills, service loans obtained without their wife's consent, and catering for emergencies. Also, they diverted some of their money to buy drinks for their friends at bars for social networking purposes. Notwithstanding, men confirmed facing many temptations from nice-looking young women, referred to as “accidents” out of the home that required money. This is what one male respondent said;

*“ …..we cannot be like women who can patiently wait for CEDO to pay them whenever they will. We have a lot of problems that need money. For us men, whenever we step out of our homes, we meet many accidents on the way. We see women wearing clothes that do not fit them (to mean skimpy dresses) and needing money. We also incur loans that our wives do not know, and we need to repay them. We cannot also leave our children to die when they are sick while we are keeping beans for people who delay buying from us."* Male respondent, FGD, Kasanda KAPUCA farmers group.

Men further emphasized the claim that women had fewer responsibilities, and even in times of need, they barely brought out their money. They keep hiding it, leaving their husbands to fall into many debts.

*“We men are the owners of homes; women are our helpers. All responsibilities at home fall on us; no one will blame a woman for a house that has collapsed. This is why women prioritize paying back to seed credit model while we prioritize school fees. I acknowledge that some men have a heart of not paying back loans"* Male FGD, Kamu Kamu group

### 4.2 Women forced to pay off husbands' seed loans

Another challenge raised in the FGDs was the unfair treatment of women in groups. Across groups, chairpersons said they had more women as most men voluntarily quit groups. The major reason men quit was shame due to failure to repay the loaned seed. When asked what the group would do to address such cases, the chairpersons, who are also men, said if these men had wives in the group, the wives would be asked to pay, and the group would automatically deduct from the wife's produce to cover the husband's debt. For chairpersons, this was the only way of recovering loaned seed and to some women covering men's debt was a sign of respect and love. This was perceived by most women as an unfair act, and taking them for granted. Women added that they did not know how their husbands spent the money they got from this secret sale of the produce. Yet, the group forced women to pay for their husbands.

*“If a husband fails to pay back as* per *the group requirement, the group automatically confiscates the wife's harvests to pay for the husband, yet each was registered as a different group member when collecting seed. Most women who were unfairly treated like this got disappointed and left the group" Female FGD, Bukuya*

Women also said that since each household member received seed independently, not as a couple, they had no obligation to repay their husband's loans if men breached the contract. This means that patriarchy at one point shaped modalities of SCM, and therefore these social and cultural barriers are likely to shape women's bias about the model. This could affect women's participation and possibly explain their declining involvement in the model. Decreased women's participation in SCM could impact their access to improved quality seeds and thus have diverse effects on food and nutrition security.

### 4.3 Land ownership, access, use, and control

Land access showed a stark gender disparity, with men enjoying greater land access (5.775 acres) compared to women (4.996 acrea), even though women constituted the most active participants in the SCM.

This unequal access to land can be attributed to prevailing social and cultural factors that determine land ownership norms within the region. Traditionally, land ownership has been predominantly male-centric, with decision-making power regarding land-related matters resting solely with men, disregarding the perspectives and opinions of women (Phiri et al., 2022). Consequently, this imbalance has become a significant cause of conflicts as land ends up being a controversial family resources that hindered active participation in the seed credit model. This is because access to land for bean production was a pre-requisite for women and men to access seeds under SCM. It also determined the quantities of seed women and men were eligible to acquire. Almost all the respondents affirmed that land for production was a big family source of conflict and that women had chosen to hire land as a solution to put an end to this conflict. For instance, male respondents in one district said they never expected any woman to have a say over land issues because they are ‘abakyala’, literally meaning they are visitors. Appreciating that land is getting scarce and the majority of households never had huge chunks of land, the little available was used by household heads who are largely men. Women who were able to hire land for bean production used money borrowed from group savings. The borrowed money was paid back after selling with minimal interest rates. However, those who could not borrow either resorted to producing beans on the small infertile piece of land allocated to them by the husband or quit participation in the SCM.

For women who insisted on utilizing the family land, their harvests were taken, and they could not question where the money had gone, leaving a sense of frustration (FGD). Those that resorted to hiring land said land was now scarce, and they had to walk long distances to get land for hire. There are more women than men who farm on hired land. One acre was said to cost UGX 200,000 per season, which was a fair price than losing their harvest or proceeds. However, some women could not afford this cost, thus lowering their participation in the SCM.

### 4.4 Limited access to family labor

In this study, most respondents were from dual households, with 97.6% headed by men. In a traditional home in the central region of Uganda, children belong to the head of the household, who is a man save for female-headed households. In some districts, women and men in FGD noted that decision-making on which farm the children went to depended on the house head's. Most women said they asked their husbands whenever they needed children to work in their gardens, and it was upon the house head to agree or decline. Every household member, including the wife, was obliged to work on the head of the house's farm until he felt he needed no more labor.

In other communities like Kiboga District, men said labor allocation was through negotiations which women denied. Women said they only used labour from children when they were free, but otherwise, child labor belonged to the household head. They added that men used family labor with a claim of shouldering all the household burdens like paying school fees, health, and looking after wives. Women contested men's allegations as false because currently, women also foot family bills. They added that when women are required to provide labor at the husband's plot as it is on theirs, they hired labor on their plots to catch up with the season. However, this increased production expenditure discouraged some women from participating in the model. Also, not being able to provide labor for their bean gardens denies women farmers the opportunity to participate fully in producing their bean grain for maximum yields.

### 4.5 Increased domestic violence in participating households

Notwithstanding the incredible benefits men and women accrued from SCM, domestic violence also shaped their participation. Some of the challenges associated with the model caused trouble for women farmers who convinced their husbands to join. For example, in case of delays from CEDO to effect payment or collect the beans harvested, men would abuse their wives for bringing seeds with no market at home. Quarrels sparked in some homes every season beans were grown, and men continued to blame women for introducing an annoying venture not capable of generating quick money. The process had bred domestic violence in some bean-growing households. One female respondent recalls how the husband mistreated her when he was stuck with his harvest for one month before CEDO collected them, yet he urgently needed the money.

“*I cannot forget how he used to quarrel with me every time he passed close to our store. He would also scold me for loitering around the village, copying and introducing to him an annoying venture that couldn't get him quick money"* female respondent, FGD, Kirangira farmers group.

Also, study results revealed that the majority of married women had experienced domestic violence when it came to spending money from their private bean plots (FGD). Those women who couldn't withstand the depression decided to withdraw their participation from the model, as echoed by their fellow women in FGDs. This denied women the opportunity to improve their lives by accessing improved quality seeds and other associated benefits.

### 4.6 Increased women's economic independence threatens men

The remarkable progress and immense benefits that women experience through their active participation in the SCM often stir feelings of insecurity among men. Consequently, some men resort to denying their wives access to crucial production resources like land and labor, resulting in disagreements within households and, in some cases, even leading to domestic violence. Some women in FGD noted that their husbands either refused them hiring land, so they used family land, usually small or infertile, leading to poor yields. Farming on a small piece of land limits women from exploring their full potential in commercial bean production. Yet literature has it that empowering women with productive resources increases their productivity (Gebre et al., 2019).

Some women reported their husbands being uncomfortable seeing them with money in their pockets. They added that some men in the community wouldn't allow their wives to participate in SCM. This was confirmed in an FGD where women in Bukuya reported that about 40% of their fellow women want to join SCM but are not supported by their husbands. Men with participating wives have either devised means of ensuring indirect control or directly controlling the use of their income. Some women reported that some men connive with health workers and fake sickness to ensure that their wives' foot medical bills and purchase special foodstuff for the sick husband.

*“For me, when my wife gets money, I die a bit so that she also spends on me, for example, buying chicken and fish until I know that the money is finished."* Male respondent, FGD, Kasanda farmers group

Other women added that when their husbands see CEDO people with a weighing scale to take their beans, they start showing them a lot of love, including fronting development programs such that wives finance them. Once men are assured that their wives' money is used up, they resume their normal way of mistreating them. To avoid domestic violence at home, some women decided to hand over some small section of their money to their husbands to keep peace at home though they reported little knowledge of what their husbands used their money for (FGD). Some men were reported to suspend their household obligations once they knew their wives had earned some income. This finding agrees with Nanyonjo et al. (2020). They noted that as women's revenue from bean production increased, men withdrew their financial support to women by forcing them to pay for household outings usually covered by husbands.

Some men confirmed the practice of assuming control over women's money during the FGD at the Kirangira farmers group and the KII with the coordinator of groups under the seed credit model as true. They said that a woman with money was hard to control and that they tried to remove some money from them whenever they sold their produce. They further said women had diverse programs for money that did not directly benefit their families, like buying expensive dresses, changing expensive hairstyles, acquiring properties, and sending money to their relatives. This evidence ties in with the finding that men intentionally ignored their monetary responsibilities to ensure their wives spent on requirements concerning all household members (Nanyonjo et al., 2020). This exercise of power by men over women's money deprives them of the opportunity to make strategic life decisions on how best to use their income to transform their living standards. As evidenced in the study, greater women's control over their income increases their agency to own assets and rent land for increased bean production (Nanyonjo et al., 2020).

## 5 Conclusion

The seed credit model as an arrangement to close the gendered seed access gap has been successful in reducing the once prevalent gender gap in seed access among smallholder common bean farmers in Uganda. The model enhanced access and availability of improved quality seed to women and men farmers with tangible benefits. Farmers who used to struggle to borrow money from friends or buy poor quality seed are now confident of access to improved quality seed. Incentives of the model include trainings and monitoring visits, enhanced participation, and the use of improved seeds. SCM increased men's participation in common bean farming which is largely considered women's crop.

Participation in the SCM was associated with economic empowerment and changing gender roles through women's enhanced skills, knowledge, and autonomy in bean production. Women were able to generate more money and reinvest in their plots to decide on off-farm management and income from the sale of crops. Women's participation was primarily shaped by gender relations and social and cultural barriers at the group and household levels. However, women's access to land and labour sometimes restricted by gender norms resulted in, women's limited access to seed, as seed credit loans were attached to available land.

Despite the potential of SCM in reducing gender gap in seed access in Uganda, women's participation in the model where their spouses were members declined over time as women were forced to repay husbands' loaned seeds because of men's unwillingness to repay loaned seeds citing heavy family responsibilities. Furthermore, the SCM is changing power dynamics in the households and some men feel threatened. As a result, domestic violence has been exacerbated as some men felt threatened by women's economic empowerment. To successfully ensure that women's economic empowerment and joint decision making is maintained, there is a need for gender education that focuses on behavioural change and gender visioning in the household. Thus, transforming the social, cultural, and power relations that shape women's and men's perceptions about and participation in the seed credit model. Only then can the full participation and benefits from SCM for women and men farmers become a reality. Lastly, there is need to make sure we don't do harm as we engage men and women to benefit and be empowered through our multiple interventions that seek to close the gender seed access gap with implications on women's income, agency, household food, and nutrition security.

## Funding

10.13039/501100008627Global Affairs Canada under the Improving Bean Production and Marketing supported this research in Africa (IBPMA) project. 10.13039/100000865Bill and Melinda Gates Foundation funded the publication of this work under the Accelerated varietal improvement and seed delivery of legumes and cereals in Africa (AVISA) projects grant number INV-009649/OPP1198373.

## Institutional review board statement

This study's ethical review and approval were waived since a government entity conducted it. Informed Consent Statement: Informed consent was obtained from all subjects involved in the study.

## Credit author statement

Grace Nanyonjo: Conceptualization, Methodology, Writing- Original draft preparation, Formal analysis and Visualization. Eileen Nchanji: Methodology, Conceptualization, Writing- Reviewing and Editing and Visualization.

## Declaration of competing interest

The authors declare no conflict of interest.

## Data Availability

Data will be made available on request.
